# Age prediction in sub-adults based on MRI segmentation of 3rd molar tissue volumes

**DOI:** 10.1007/s00414-023-02977-4

**Published:** 2023-02-22

**Authors:** Mai Britt Bjørk, Sigrid Ingeborg Kvaal, Øyvind Bleka, Tomas Sakinis, Frode Alexander Tuvnes, Mari-Ann Haugland, Peter Mæhre Lauritzen, Heidi Beate Eggesbø

**Affiliations:** 1 Institute of Clinical Dentistry, Faculty of Dentistry, University of Oslo, Postboks 1109, Blindern, N-00317 Oslo, Norway; 2 Department of Forensic Sciences, Oslo University Hospital, Postboks 4950 Nydalen, OUS, Rikshospitalet, 0424 Oslo, Norway; 3 Division of Radiology and Nuclear Medicine, Oslo University Hospital, Postboks 4950 Nydalen, OUS, Ullevål, 0424 Oslo, Norway; 4 Faculty of Health Sciences, Department of Life Sciences and Health, Oslo Metropolitan University, Postboks 4, St. Olavs plass. 0130, Oslo, Norway; 5 Department of Radiology and Nuclear Medicine, Institute of Clinical Medicine, Faculty of Medicine, University of Oslo, Postboks 4950 Nydalen, OUS, Rikshospitalet, 0424 Oslo, Norway

**Keywords:** Age estimation, Sub-adults, Third molar, Magnetic resonance imaging, Segmentation

## Abstract

**Purpose:**

Our aim was to investigate tissue volumes measured by MRI segmentation of the entire 3rd molar for prediction of a sub-adult being older than 18 years.

**Material and method:**

We used a 1.5-T MR scanner with a customized high-resolution single T2 sequence acquisition with 0.37 mm iso-voxels. Two dental cotton rolls drawn with water stabilized the bite and delineated teeth from oral air. Segmentation of the different tooth tissue volumes was performed using SliceOmatic (Tomovision^©^). Linear regression was used to analyze the association between mathematical transformation outcomes of the tissue volumes, age, and sex. Performance of different transformation outcomes and tooth combinations were assessed based on the *p* value of the age variable, combined or separated for each sex depending on the selected model. The predictive probability of being older than 18 years was obtained by a Bayesian approach.

**Results:**

We included 67 volunteers (F/M: 45/22), range 14–24 years, median age 18 years. The transformation outcome (pulp + predentine)/total volume for upper 3rd molars had the strongest association with age (*p* = 3.4 × 10^−9^).

**Conclusion:**

MRI segmentation of tooth tissue volumes might prove useful in the prediction of age older than 18 years in sub-adults.

## Introduction

Several legal issues necessitate determination of an individual’s age either because the given age is doubtful or the chronological age is withheld or unknown. The age of a perpetrator or a victim may affect sentencing. Even in sports, age estimation has become necessary in order to avoid fraud, maintain fairness, and protect the health of athletes [1].

Depending on the situation, different age limits are applicable, ranging from 12 to 21 years. However, age estimation is most frequently applied in asylum cases, where those aged younger than 18 years may have certain rights [2, 3]. According to the definition of UN, “A child means every human being below the age of eighteen years unless under the law applicable to the child, majority is attained earlier” [3].

Age estimation of children is normally performed by some kind of grading or measurements of the development from childhood to an adult fully grown person. Growth and development are measured by height, weight, sexual maturity, dental and skeletal development, and psychological maturations, all traits are with normal biological variation [4].

Skeletal age estimation in children and sub-adults are commonly graded from the development and closure of the bony metaphysis. The atlas with radiographs of hand/wrist development by Greulich and Pyle was published in 1959 to document the normal skeletal development of children with known age. Since then, this atlas has been the most common method for skeletal age estimation by plain radiograph [5, 6]. However, the validation of this reversed application of the atlas has shown 95% prediction intervals of around 4 years after 10 years of age [7].

In dental age estimation of sub-adults, the 3rd molars are most frequently used as it is the last tooth to complete development. Staging of the 3rd molars, according to Demirjian et al., has the best scientific documentation [8, 9].

Several radiological modalities have been applied in age estimation, most notably plain radiography, orthopantomogram (OPG), computed tomography (CT), cone beam computed tomography (CBCT), and magnetic resonance imaging (MRI).

The first paper of skeletal MRI in age estimation was published in 1992 and dental MRI in 2015 [10–12]. In recent years, MRI has gained momentum, since radiation-free procedures are preferable, especially in children and sub-adults [13].

The International Olympic Committee acknowledged the importance of MRI for age estimation in sports in their 2010 consensus paper [1, 14].

Although no consensus exists on the choice of method, some studies report improved precision of age estimates by a combination of two or more independent physical traits, and this is recommended for legal purposes [15, 16]. BioAlder, which is built on large, global datasets, is one of few tools complying with this recommendation by combining grading of mandibular left 3rd molar on OPG, and left hand/wrist bones on radiographs according to Greulich and Pyle [5, 8, 13, 17].

Measurements of molar tissue have been explored as a possible means of increasing precision of dental age estimates compared to staging according to Demirjian [8]. OPG, CBCT, and MRI have been employed, but measurements have mostly been linear and only included the tooth crown, and not including tissue volumes of the entire molar due to the complex root anatomy [18–20]. The anatomy of 3rd molars has been described as unpredictable, where one root has unusual morphology, with the number of root canals varying from one to six [20].

Hence, there might be an added value of measuring volumes of the entire molar, and apply different combinations of measurements from the four quadrants. To our knowledge, this has not been done previously.

Our aim was to investigate the entire 3rd molar’s tissue volumes by MRI segmentation for prediction of a sub-adult being older than 18 years old.

## Material and method

The study was approved by the *Data Protection Officer* (PVO), Oslo University Hospital, and performed in accordance with the Declaration of Helsinki [21]. All participants signed a declaration of consent, and those who were younger than 17 years got approval from parents or legal guardians.

### Participants

We enrolled 99 healthy volunteers. The age (in days) and sex of the participants was registered. Inclusion criteria were age from 14 to 24 years with no contraindications according to the MRI check list from The Norwegian Directorate of Health 2017.

The participants were recruited from sports clubs and universities in the period 2018–2021. The median age was 18 years. Female (F):65, male (M):34.

### MRI acquisition

All MRI examinations were performed using a 1.5-T scanner (Avantofit, Siemens, Erlangen, Germany) using a bilateral surface coil (Head Neck 20 and Flex Small 4 used in combination).

Our acquisition had a scan time of 5 min and 4 s and yielded 0.37 mm iso-voxels, in which a volume of 1 ml (roughly equivalent to one tooth) consists of 20,000 voxels.

Scan parameters are displayed in Table 1.

**Table 1:** Acquisition parameters for the MRI sequence

Sequence	Time	Voxel size/reconstructed voxel size (mm)	Resolution	FOV (mm)	Number of slices	TR (ms)	TE/effective TE (ms)	Averages	Slabs number	Flip angle
3D TSE	05:04	0.74 × 0.74 × 0.74/0.37 × 0.37 × 0.37	256 × 256	190	192	1400	182/80	1.4	1	T2 var

*TSE* turbo spin echo, *FOV* field of view, *TR* time of repetitions, *TE* echo time

### Bite material

**Fig. 1:**
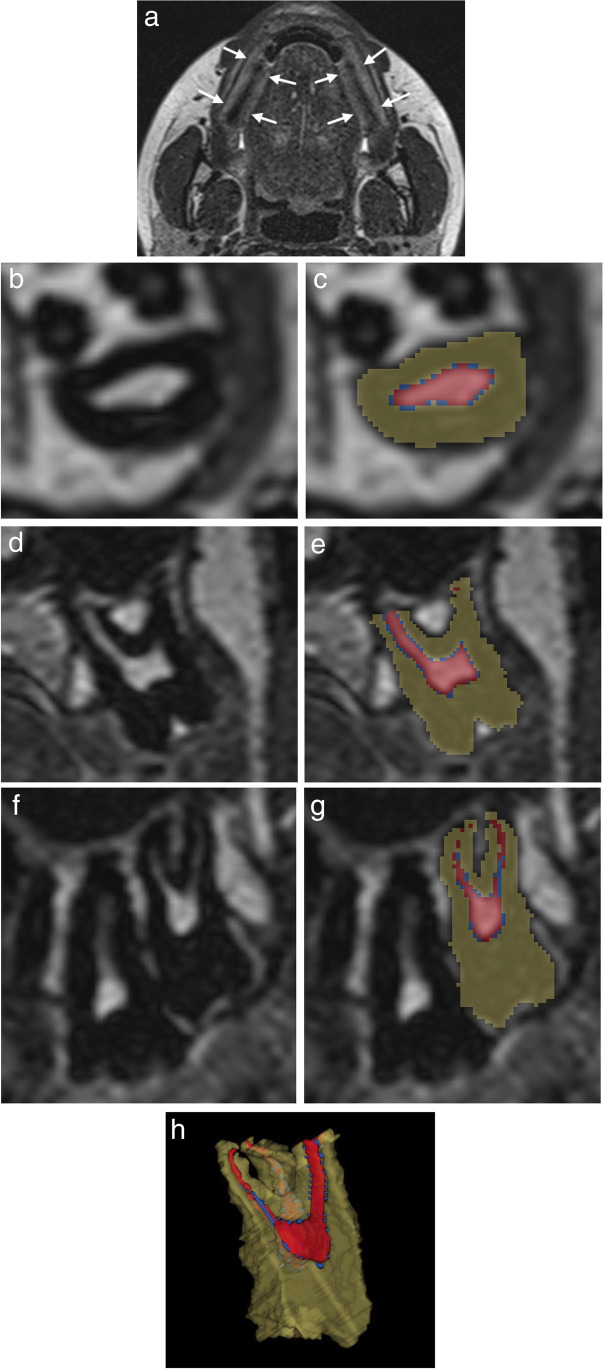
**a** Axial MRI shows correct bilateral placement of dental cotton rolls filled with water (arrows) between the molars. The cotton rolls delineated the upper and lower molars and stabilized the bite. **b**, **c** Axial MRI through the upper jaw shows unsegmented and segmented tooth 28. Green for hard tooth tissue, blue for predentine, and red for pulp. **d**, **e** Coronal MRI through the upper jaw shows unsegmented and segmented tooth 28. Green for hard tooth tissue, blue for predentine, and red for pulp. **f**, **g** Sagittal MRI through the upper jaw shows unsegmented and segmented tooth 28. Green for hard tooth tissue, blue for predentine, and red for pulp. **h** 3D rendering of the segmentation, with a wedge of approximately one quarter removed to visualize the pulp. Green for hard tooth tissue, blue for predentine, and red for pulp

Two cotton rolls size 2 filled with 2 ml of water were placed bilaterally between the molars in order to displace air for better delineation of the teeth, and to stabilize the bite, as shown in Fig. 1a.

### Segmentation

The MRI examinations were separated into upper (maxillary) and lower (mandibular) teeth. Semi-automated segmentation of three different tooth tissues of the 3rd molars, 18 (upper right), 28 (upper left), 38 (lower left), and 48 (lower right), was performed using SliceOmatic Tomovision^©^, Canada.

Dentine, enamel, and cementum could not be differentiated based on T2 signal intensity in our MRI sequence. These tissues were segmented collectively and are referred to as hard tooth tissue. Segmentation of three tooth tissues pulp, predentine, and hard tooth tissue, was performed on axial images based on T2 signal intensity thresholding, as shown in Fig. 1b–h. Lower and upper thresholds were set at 0 and 63 for hard tooth tissue, 64 and 100 for predentine, and ≥ 101 for pulp, which we previously experienced to match these tissues on tooth ground sections. In order to agree on the teeth delineation and separation from surrounding tissues, a ground truth segmentation was established for the first five participants by two experienced forensic dentists and an experienced head and neck radiologist in consensus. The apical end of a root was defined as the point where tooth hard tissue could be identified on at least two sides, and segmentation was not performed beyond this point.

The volume of each segmented tooth tissue was calculated in ml.

### Statistical analyses

The association between explanatory variables (age and sex) and response variables (the three tooth tissue volumes, pulp, predentine, and hard tooth tissue) were analyzed with linear regression models. We used the natural logarithm (ln) of the tooth tissue volumes in order to achieve linearity and simplify the statistical analysis.

### Transformation and Pearson correlation coefficient

In order to find the best response variable, we explored four different transformations of the tooth tissues, resulting in 10 outcomes as shown in Table 2.

**Table 2:** Four transformations (**1–4**) of the tooth tissue volumes resulting in 10 outcomes (1 and 2–4a–c)

**1. Total: pulp + predentine + hard tooth tissue**		
**2. *x*/(total − *x*)**	**3. *x*/total**	**4. (*x* + *y*)/total**
(a) Pulp/(total − pulp)	(a) Pulp/total	(a) (Pulp + predentine)/total
(b) Predentine/(total − predentine)	(b) Predentine/total	(b) (Pulp + hard tooth tissue)/total
(c) Hard tooth tissue/(total − hard tooth tissue)	(c) Hard tooth tissue/total	(c) (Predentine + hard tooth tissue)/total

*x* and *y* are either pulp, predentine, or hard tooth tissue shown in outcomes a–c for the transformations 2–4

The 10 outcomes were assessed with Pearson correlation. Overlapping transformations were defined as *R* ≥ 0.999. Outcomes 2a and 2b overlapped with 3a and 3b, and outcome 2c overlapped with 4a. Hence, outcomes 2a–c were deemed redundant and not included in further statistical analysis.

### Regression analysis exploration

A large number of regression models were explored incorporating the seven remaining transformation outcomes, different combinations of the four 3rd molars, different models of age and sex, and different weighting of model variance.

### Selection of models of age and sex and variance weighting

Age was always included as an exploratory variable in the regression model. The variable sex was incorporated into the model in five different ways: (i) sex not considered; (ii) different intercepts for sex; (iii) different age slopes for sex (but common intercept); (iv) different age slopes and intercept for sex; (v) separate model for the two sexes (same as iv but also different variance).

We also explored three different weighting of the variance models: either as constant (default = 1), age or (1/age).

Akaike information criterion (AIC) was used to select the model type for sex and variance weighting.

### Selection of tooth combinations and transformation outcomes

The 3rd molars were analyzed separately, and as the average in the following seven combinations: (18 and 28), (38 and 48), (18 and 48), (28 and 38), (18 and 38), (28 and 48), and (18, 28, 38, and 48).

Performance of the transformation outcomes and tooth combinations were based on the *p* value of the age variable, common or separate for each sex depending on the selected model.

The best transformation outcome and tooth combination were selected based on lowest *p* values. The *p* value incorporates both the steepness of the slope and data variation. The lower the *p* value, the better the method is for age prediction.

### Age prediction by Bayesian approach

The selected best performing model from the exploration was used for age prediction.

We used a Bayesian approach to describe the uncertainty of an individual’s age [22, 23]. A prior uniform age distribution was defined from 14.0 to 23.0 years.

Posterior distribution of age after applying Bayes theorem for the transformation outcome, in each selected tooth combination was used to estimate the probability for being older than 18 years, in each sex.

The analysis was conducted using *R v4.0.2*. The regression model was performed using the *lm* and *predict* functions.

## Results

From the 99 enrolled volunteers, 32 were excluded due to missing 3rd molars (*n* = 4), tooth unsuitable for segmentation (*n* = 15), movement artifacts (*n* = 7), and incorrect use of the dental cotton rolls (*n* = 6).

**Fig. 2:**
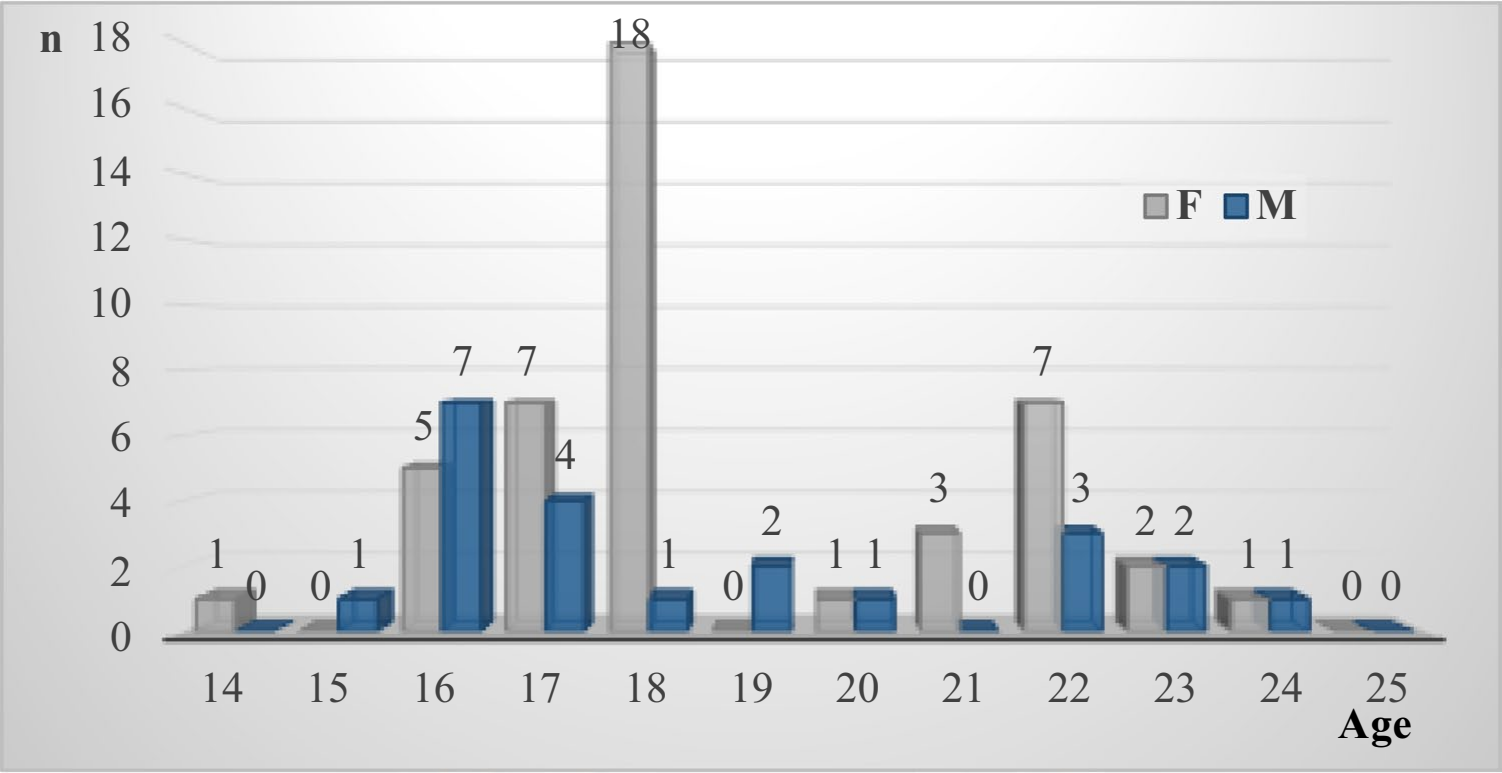
Age distribution of 67 healthy volunteers 44 females (F) and 23 males (M)

After exclusion, we had 67 volunteers, 45 females, 22 males, with median age 18 years (range 14–24 years). The age distribution is shown Fig. 2.

### Tooth tissue volumes

**Fig. 3:**
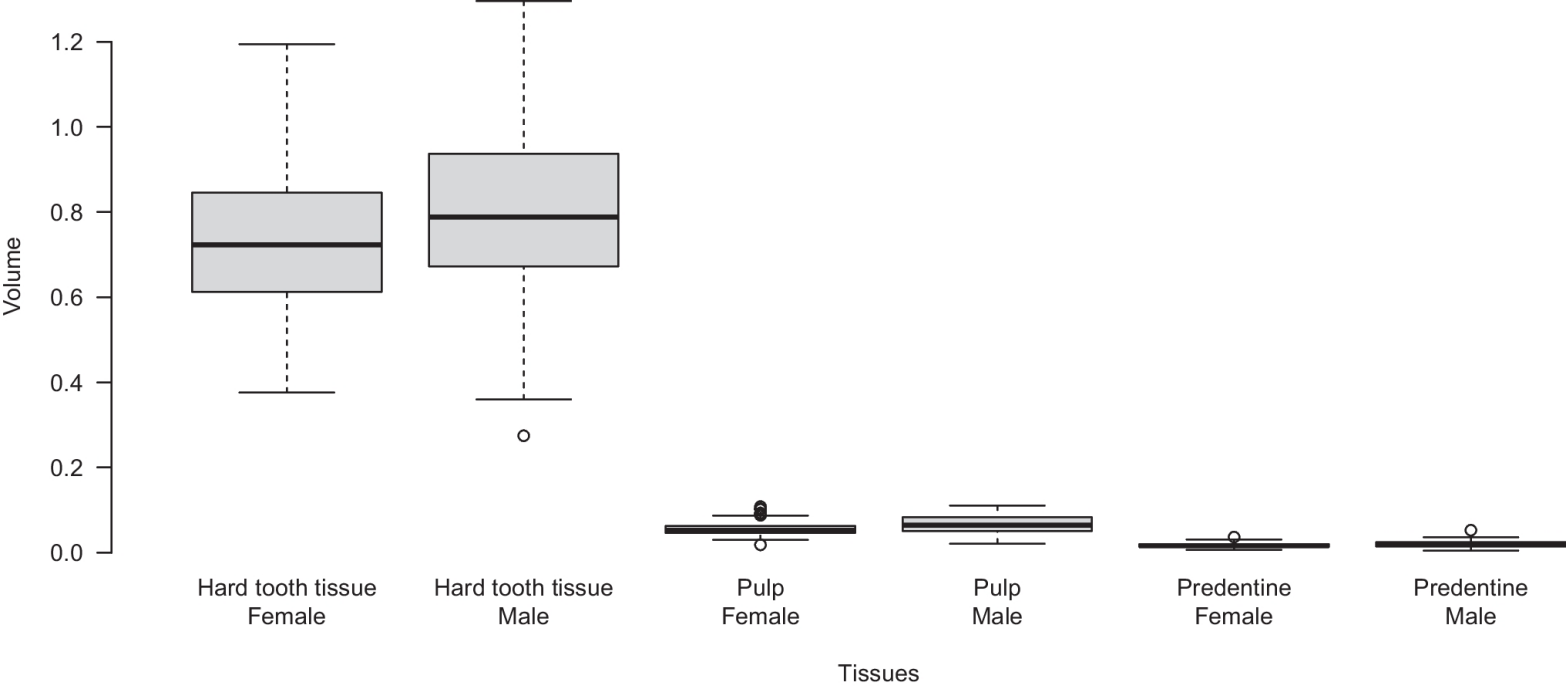
Tissue volumes for pulp, predentine, and hard tooth tissue for each sex. The box represents the upper and lower quartiles, the vertical thick lines the median, and the vertical thin lines of the whiskers represent maximum and minimum. Outliers are marked as circles

The median volumes of hard tooth tissue, pulp, and predentine were as follows: 0.712, 0.052, and 0.016 ml for females and 0.788, 0.065, and 0.02 ml for males. The distribution is shown in Fig. 3.

### Selected model

Transformation outcome 4a, (pulp + predentine)/total, performed best. Applied to this transformation outcome, the best tooth combination was the upper 3rd molars for both sexes (*p* = 3.4 × 10^−9^). For this selection, the regression model with the sum of age and sex, as intercept and the variance weighting as ratio equals 1/age performed best.

**Fig. 4:**
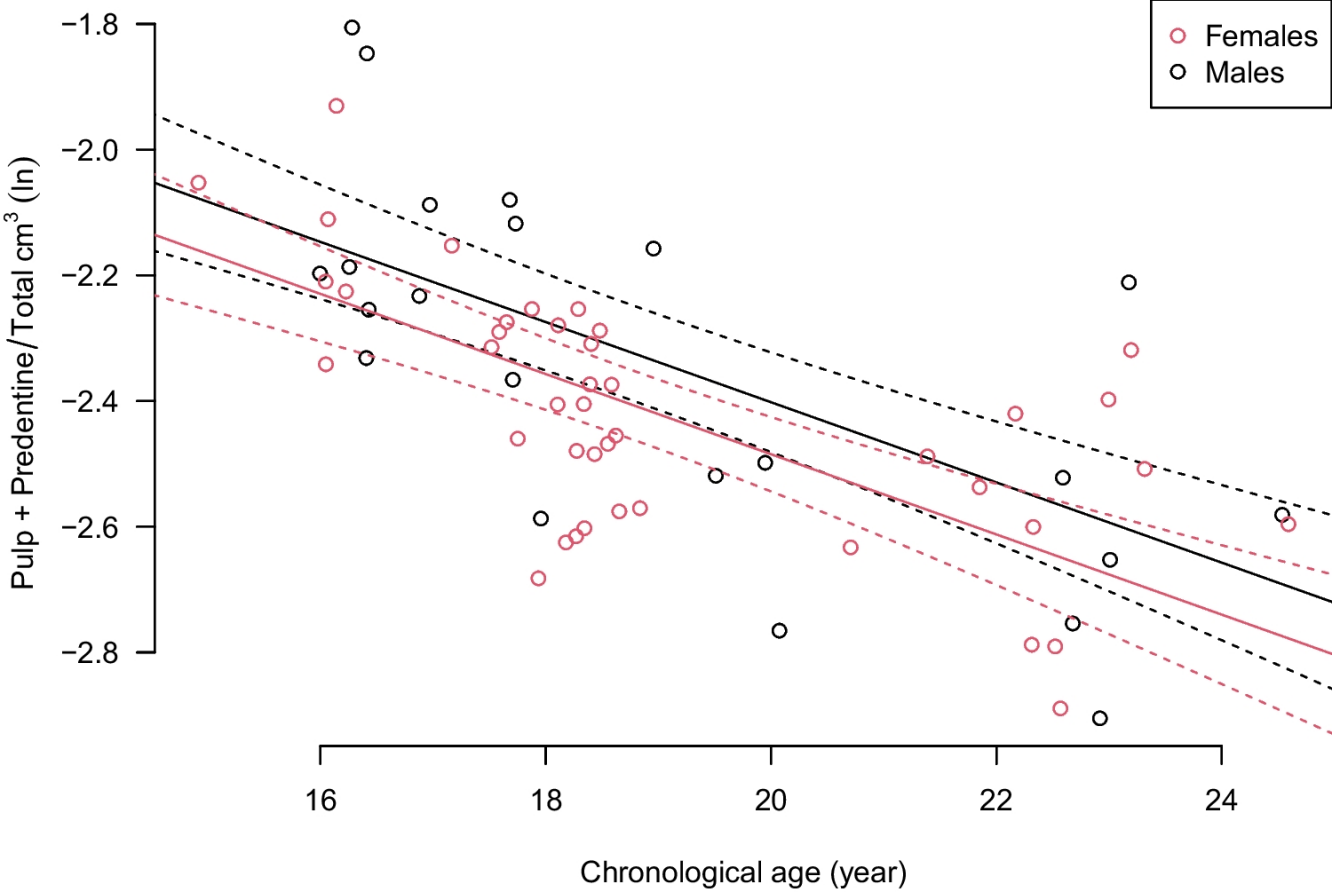
The regression model with the response variable natural logarithm of the ratio (pulp + predentine)/total applied to the upper 3rd molars for both sexes (*p* = 3.4 × 10^−9^) on *y*-axis against chronological age as explanatory variable on *x*-axis. The expectations are shown as solid lines and 95% confidence intervals as dashed curves (red for females, black for males). The sexes have the same slope but different intercept. Observed data are shown as circles

Estimated age trend with 95% confidence interval for this model, which was selected for age prediction, as shown in Fig. 4. This model is henceforth referred to as “the selected model.”

Best single 3rd molar was tooth 18 in males (*p* = 1.0 × 10^−7^) followed tooth 28 in females (*p* = 1.9 × 10^−7^). However, these teeth were not selected for further analysis.

### Age prediction by Bayesian approach

Four hypothetical observations were placed in uniform intervals between the actual minimum and maximum observations. For the selected model, these hypothetical observations were used as examples to illustrate the probability of an individual being older than 18 years.

**Fig. 5:**
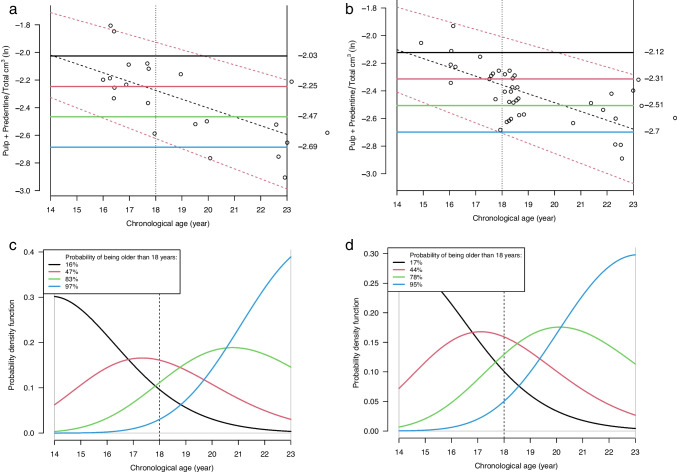
**a**–**d** The expectation (dashed black oblique line) for males (**a**) and females (**b**) and 95% prediction interval (dashed red oblique lines) of the natural logarithm of (pulp + predentine)/total applied to the upper 3rd molars (*y*-axis) against chronological age (*x*-axis). The prior age distribution (14.0–23.0 years) is shown as solid vertical black lines. The 18-year threshold is shown as a vertical dashed line. The color coded solid horizontal lines in black, red, green, and blue illustrate four different ratios of (pulp + predentine)/total, from the natural logarithm of hypothetical measurements in individuals. The posterior age distributions for males (**c**) and females (**d**), after applying Bayes theorem. The age distribution curves are color-coded (black, red, green, and blue) and correspond to the hypothetical ratios in **a** and **b**. The probabilities of being older than 18 years for each ratio are shown in the legends. The area under each curve is 100% of all probabilities (area equal to 1). For the blue curve, the probability for being older than 18 years is 97% for males taken the corresponded hypothetical blue color-coded vertical line in **a**. The age distribution curves are limited by the prior age distribution (14.0–23.0 years). The 18-year threshold is shown as a vertical dashed line

The age expectation with 95% prediction interval (2SD) for the individual based variation conditioned on age as shown in Fig. 5a, b.

In males, these four hypothetical observations were 0.068, 0.085, 0.11, and 0.13 giving 97%, 87%, 47%, and 16% possibility of being older than 18 years, as shown in Fig. 5c.

In females, these four hypothetical observations were 0.067, 0.082, 0.099, and 0.12 giving 95%, 78%, 44%, and 17% possibility of being older than 18 years, as shown in Fig. 5d.

## Discussion

In this study, we have developed a dedicated in vivo MRI protocol for the 3rd molars with high spatial resolution and short acquisition time and a method for segmentation of the entire tooth volumes, with three tooth tissues hard tooth tissue, pulp and predentine. Further, we have shown a very strong correlation between tooth tissue development and age and established a model to predict the probability of being older than 18 years.

### MRI

To our knowledge, 1.5-T MRI has not been used in dental age estimation before, and our study shows that such acquisitions may be used for tooth tissue segmentation. While previous studies have used a 3-T MRI [24–27] and focused on apical closure of the 3rd molar root or linear measurements, we used segmentation to measure tooth tissue volumes.

A previous study has proposed 6:30 min as a clinically acceptable acquisition time [28]. Our single-sequence high-resolution acquisition with 0.37 mm iso-voxels had an acquisition time of 5 min 4 s. Hence, with a shorter acquisition time, we accomplished a comparable resolution to previous studies using 0.33 × 0.33 × 2 mm^3^ T2 FSE or 0.59 × 0.59 × 1 mm^3^ T1 3D FSE. A further increase in resolution to 0.3 mm iso-voxels would have increased the scan time to 7 min 13 s [26]. We used dental cotton rolls filled with water to displace air for better delineation of the teeth and to stabilize the bite while previous studies have performed MRI with or without a bite bar [26].

### Segmentation

Although dentine, enamel, and cementum cannot be distinguished on regular MRI acquisitions, we have shown that a distinction can be made between tooth hard tissue, predentine, and pulp, and used in segmentation of the whole 3rd molar teeth. We did not histologically confirm the tissue segmentations in this cohort, but we have previously experienced that the tissue volumes segmented with this method correspond to hard tooth tissue, predentine, and pulp on ground sections. Further, the strong correlation with age confirm that the segmentations correspond to developing tissues.

Two studies including extracted teeth, using a 9.4-T ultrashort echo time MRI, managed to determine pulp cavity volume, but the field of view and spatial resolution had to be adjusted for each different type of tooth [29, 30].

Other studies using CBCT on 1st and 2nd molars have set the pulp chamber floor as the “cut off plane” and excluded the roots [18]. Volume of the coronal tooth pulp chamber was calculated in order to avoid the complex multi-rooted system.

### Results

Teeth are appropriate for age prediction since their age related changes are highly resistant to time, environmental, hormonal, nutritional, and physical impact [31]. Dentine, which surrounds the pulp, is continuously deposited throughout life. The pulp volume is decreasing with advancing age [11]. Once the tooth is fully formed, the size of the tooth does not change. The pulp ratio has been shown to be an appropriate variable in age estimation [32].

The 3rd molars are the only teeth still developing after 16 years of age and most have completed their development in the early twenties. Epiphyseal gaps of hand/wrist bones close around the age of 18 years, the clavicles at 21 years [27]. This support why 3rd molars can be important in estimating the age to be older or younger than 18 years [15].

Although a previous study reported that the maxillary 2nd molar was best suited for age estimation based on pulp chamber/cavity volume [18], we decided to focus on the 3rd molars, since this molar presents the greatest variation in morphology and development [20, 33]. Using 3D imaging, which presented the entire tooth, we found that the upper molars performed best. This is interesting since the upper molars have been studied less with conventional 2D images like OPG, since they are frequently angulated and/or superimposed on adjacent structures [34].

Admittedly, our method is vulnerable to agenesis of one or more of the 3rd molars. The rate of which ranges from 5 to 56% in different studies, and being more frequent in the maxilla and among females [35]. When there is agenesis of one or more 3rd molars, delays in development must be considered, and our results may not be valid in this group [36].

Combinations with other anatomical sites (other teeth, hand, and clavicle) may reduce the problems caused by missing 3rd molars [27].

### Transformation outcome

We explored four different transformations of the tooth tissues, resulting in 10 outcomes. Of these, the ratio of (pulp + predentine)/total had the strongest correlation with age. This applied to each sex separately and pooled. This result is difficult to compare to previous studies that have used other modalities, segmentation of the crown only, linear measurements, tooth contours or root closure [24–27]. We have not been able to identify any in vivo studies using tissue volumes from the entire tooth.

### Sex

Males had the best results in our study compared to females in the pulp chamber study [18]. Another study using pulp chamber volume of the upper 2nd molars had best results in males compared to females [33].

Including sex for age estimations should be recommended and it is in agreement with other studies [18, 33, 37, 38]. This is in contrast to some studies where other teeth were studied and concluded that sex made no difference [39, 40].

### Statistical analysis

In contrast to regression analysis which frequently overestimates young individuals and underestimates elderly [41], the Bayesian model optimize the evidence evaluation [42].

The Bayesian approach also avoids the age mimicry issue [22, 23] but requires the specification of a prior age distribution. We set the lowest age prior to 14 years since the 3rd molar is not suitable for measurements below this age, and our youngest participant was almost 15 years old.

The lower age prior in this study was given by 3rd molar development, while the upper prior was a matter of choice. Increasing the upper prior of the model will increase the risk of falsely classifying a person as older than 18 years. Conversely, decreasing the upper prior will increase the risk of falsely classifying a person as younger than 18 years. In our study, the upper prior was set at 23 years, while BioAlder set the upper prior at 20.5 years for dental assessment.

### Limitations

This study may have few participants, and there is limited ethnic variation in the study population, and the total absence of caries imply a limited variation in socioeconomic status. It is uncertain how the model will perform in other ethnic groups [43]. Although we have shown a strong association between 3rd molar tissue volumes and age, our results have not been validated by an independent cohort. Whether our method may contribute to narrowing the prediction intervals for chronological age, alone or in combination with other teeth or measurements like DNA methylation [44], remains to be seen.

## Conclusion

MRI segmentation of tooth tissue volumes might prove useful in the prediction of age older than 18 years in sub-adults.

## Data Availability

All data was registered, including data that was deleted or changed. Anonymized data was exported for statistical calculations. After database lock, the data was saved according to current regulation.

## Abbreviations

AIC: Akaike information criterion
CBCT: Cone beam computed tomography
CT: Computed tomography
DCNN: Deep convolutional neural network
FOV: Field of view
FSE: Fast spin echo
MRI: Magnetic resonance imaging
OPG: Orthopantomogram
TE: Echo time
TR: Repetition time
TSE: Turbo spin echo
T2: Transverse relaxation time
18: Upper right 3rd molar
28: Upper left 3rd molar
38: Lower left 3rd molar
48: Lower right 3rd molar
